# Prognostic and Predictive Value of DAMPs and DAMP-Associated Processes in Cancer

**DOI:** 10.3389/fimmu.2015.00402

**Published:** 2015-08-07

**Authors:** Jitka Fucikova, Irena Moserova, Linda Urbanova, Lucillia Bezu, Oliver Kepp, Isabelle Cremer, Cyril Salek, Pavel Strnad, Guido Kroemer, Lorenzo Galluzzi, Radek Spisek

**Affiliations:** ^1^Sotio, Prague, Czech Republic; ^2^Department of Immunology, 2nd Faculty of Medicine, University Hospital Motol, Charles University, Prague, Czech Republic; ^3^Equipe 11 labellisée par la Ligue Nationale contre le Cancer, Centre de Recherche des Cordeliers, Paris, France; ^4^U1138, INSERM, Paris, France; ^5^Sorbonne Paris Cité, Université Paris Descartes, Paris, France; ^6^Université Pierre et Marie Curie, Paris, France; ^7^Metabolomics and Cell Biology Platforms, Gustave Roussy Comprehensive Cancer Institute, Villejuif, France; ^8^Equipe 13, Centre de Recherche des Cordeliers, Paris, France; ^9^Institute of Hematology and Blood Transfusion, Prague, Czech Republic; ^10^Department of Gynecology and Obsterics, 2nd Faculty of Medicine, University Hospital Motol, Charles University, Prague, Czech Republic; ^11^Pôle de Biologie, Hopitâl Européen George Pompidou, AP-HP, Paris, France; ^12^Gustave Roussy Comprehensive Cancer Institute, Villejuif, France

**Keywords:** ATP, autophagy, calreticulin, ER stress response, HSPs, type I interferon

## Abstract

It is now clear that human neoplasms form, progress, and respond to therapy in the context of an intimate crosstalk with the host immune system. In particular, accumulating evidence demonstrates that the efficacy of most, if not all, chemo- and radiotherapeutic agents commonly employed in the clinic critically depends on the (re)activation of tumor-targeting immune responses. One of the mechanisms whereby conventional chemotherapeutics, targeted anticancer agents, and radiotherapy can provoke a therapeutically relevant, adaptive immune response against malignant cells is commonly known as “immunogenic cell death.” Importantly, dying cancer cells are perceived as immunogenic only when they emit a set of immunostimulatory signals upon the activation of intracellular stress response pathways. The emission of these signals, which are generally referred to as “damage-associated molecular patterns” (DAMPs), may therefore predict whether patients will respond to chemotherapy or not, at least in some settings. Here, we review clinical data indicating that DAMPs and DAMP-associated stress responses might have prognostic or predictive value for cancer patients.

## Introduction

For a long time, tumors were considered as highly homogenous entities resulting from the clonal expansion of a single cell with specific genetic or epigenetic defects (1). Now, it is clear that both hematopoietic and solid neoplasms are highly heterogenous, not only because malignant cells with distinct phenotypic and behavioral features generally co-exist, but also because multiple non-transformed cells are co-opted by growing cancers to support their needs. This is especially true for solid tumors, which contain an abundant non-malignant cellular compartment encompassing stromal, endothelial, and immune components (2, 3). The immune compartment of the tumor mass is *per se* very heterogenous, varying not only with tumor type, stage, and therapeutic regimen, but also on an inter-individual basis (4). Evidence accumulating over the last decade indicates indeed that human tumors form, progress, and respond to therapy in the context of an intimate, bidirectional interaction with the immune system (5, 6). Thus, clinically manifest neoplasms can develop only when they are able to escape immunosurveillance (7, 8), and they do so by evolving under the selective pressure imposed by the immune system (6, 9). Moreover, the composition, density, and intratumoral localization of the immune infiltrate have been ascribed with a robust prognostic or predictive value in several cohorts of cancer patients (10–12). Finally, the efficacy of most, if not all, therapeutic regimens commonly employed in cancer patients has been etiologically linked to the (re)elicitation of an adaptive immune response targeting malignant cells (13, 14).

Conventional chemotherapeutics and targeted anticancer agents can favor the (re)elicitation of anticancer immune responses through several mechanisms (13–15). A precise description of all these immunostimulatory pathways goes largely beyond the scope of this review, and can be found in Ref. (13, 14). However, it is useful to note that anticancer therapy can boost immunosurveillance by either of two mechanisms. First, it can directly modulate the functions of immune cells, including dendritic cells (DCs), myeloid-derived suppressor cells (MDSCs), tumor-associated macrophages (TAMs), CD8^+^ cytotoxic T lymphocytes (CTLs), and CD4^+^CD25^+^FOXP3^+^ regulatory T (T_REG_) cells (14). Second, it can promote the immunogenicity or adjuvanticity of cancer cells as it subjects them to a state of stress (which sometimes leads to their death) (14, 16). In particular, some chemotherapeutic agents like anthracyclines, oxaliplatin, and bortezomib, as well as specific forms of radiation therapy and photodynamic therapy, are able to trigger a functionally peculiar variant of caspase-dependent cell death that *per se* is perceived as immunogenic by the immune system (17–21). This means that, upon inoculation in immunocompetent hosts, cells succumbing to such an immunogenic form of cell death are sufficient to elicit an adaptive immune response against dead cell-associated antigens associated with the establishment of immunological memory (22, 23).

Mechanistically, immunogenic cell death (ICD) relies on the pre-mortem activation of several stress response pathways that are associated with the emission of a well-defined set of danger signals by dying cancer cells (24–26). When delivered in the correct spatiotemporal order, such damage-associated molecular patterns (DAMPs) recruit specific cellular components of the innate and adaptive immune system to the tumor bed and activate them, ultimately resulting in the elicitation of a tumor-targeting immune response (22, 26). Conversely, in physiological conditions DAMPs are generally inaccessible to the immune system, and serve metabolic, structural, or enzymatic functions (26–28). Of note, DAMPs are not only involved in ICD-associated anticancer immunosurveillance, but also play a key role in the etiology of shock conditions triggered by trauma and other non-microbial stimuli (29, 30).

So far, four DAMPs have been ascribed a non-redundant, essential function in the context of anthracycline-induced ICD, namely (1) the pre-apoptotic exposure of the endoplasmic reticulum chaperone calreticulin (CALR) and various heat-shock proteins (HSPs) on the outer leaflet of the plasma membrane, which ensues the activation of an ER stress response orchestrated around the phosphorylation of eukaryotic translation initiation factor 2A, 65 kDa (EIF2A) and the overgeneration of reactive oxygen species (ROS) (31–36); (2) the production of type I interferons (IFNs), which depends on Toll-like receptor 3 (TLR3) signaling (37–40); (3) the secretion of ATP, which relies on the activation of autophagy (41, 42); and (4) the release of the non-histone chromatin-binding protein high mobility group box 1 (HMGB1) into the extracellular space, which correlates with cell death induction (43, 44). The role of other DAMPs such as mitochondrial DNA (mtDNA), *N*-formylated peptides, cardiolipin, and filamentous (F)-actin in ICD signaling has not yet been investigated in detail (30, 45).

Accumulating preclinical evidence indicates that monitoring DAMPs or DAMP-associated stress responses in cancer patients may have prognostic or predictive value. Here, we review clinical data lending further support to this hypothesis.

## Calreticulin, HSPs, and the ER Stress Response

Cancer cells undergoing ICD exhibit several manifestations of the so-called unfolded protein response (UPR) (34, 46), i.e., the ensemble of mechanisms aimed at the re-establishment of intracellular homeostasis following the accumulation of unfolded proteins within the ER lumen (47). In particular, ICD is etiologically associated with the phosphorylation of EIF2A on S51 (48), and this appears to be required for the exposure of CALR and HSPs on the surface of dying cells (34). On the cell surface, CALR, heat shock 70 kDa protein 1A (HSPA1A, best known as HSP70) and heat shock protein 90 kDa alpha (cytosolic), class A member 1 (HSP90AA1, best known as HSP90) play partially overlapping (but not identical) immunostimulatory functions. Indeed, CALR, HSP70 and HSP90 all bind to low density lipoprotein receptor-related protein 1 (LRP1, best known as CD91) on antigen-presenting cells (APCs), hence stimulating the uptake of dead cell-associated antigens in the form of apoptotic bodies (32, 33). HSP70 and HSP90 favor CTL cross-priming by APCs upon interaction with Toll-like receptor 4 (TLR4) and CD14 (33, 49–51). In some settings, soluble HSPs and CALR operate as cytokines, stimulating the NF-κB-dependent secretion of pro-inflammatory mediators like interleukin-6 (IL-6) and tumor necrosis factor α (TNFα) (52, 53). HSP70 boosts the cytotoxic functions of natural killer (NK) cells by binding to killer cell lectin-like receptor subfamily D, member 1 (KLRD1, best known as CD94) (54, 55). Moreover, ecto-HSP70 binds to phosphatidylserine (PS), a phospholipid that is exposed in the course of regulated cell death owing to the caspase-dependent activation of phospholipid scramblase 1 (PLSCR1) (56). The actual relevance of this interaction for ICD, however, has not been determined yet. Along similar lines, it remains obscure whether additional CALR receptors such as CD69; thrombospondin 1 (THBS1); complement component 1, q subcomponent (C1q); lectin, mannose-binding, 1 (LMAN1); and various integrins of the CD49 family are etiologically implicated in the perception of ICD (57). Of note, ecto-CALR has been suggested to act as a DC receptor for the tumor-associated antigen (TAA) NY-ESO-1, hence facilitating the interaction between DCs and malignant cells (58). To the best of our knowledge, however, this finding has not been confirmed by independent investigators.

Accumulating clinical evidence indicates that various parameters linked to ICD-associated CALR and HSP signaling may have prognostic or predictive value for cancer patients (Table 1). In addition, the results of multiple clinical trials suggest that HSPs can be harnessed as a means to boost the efficacy of anticancer vaccines. High CALR levels in malignant cells have been shown to correlate with favorable disease outcome in a cohort of 68 neuroblastoma patients (irrespective of treatment) (59), and in a cohort of 23 lung cancer patients and 220 ovarian cancer patients treated with ICD inducers (*i.e*., radiotherapy and paclitaxel, respectively) (60). Moreover, increased CALR expression by cancer cells has been associated with tumor infiltration by CD45RO^+^ memory T cells and improved 5-year overall survival amongst 68 subjects with Stage IIIB colorectal carcinoma (CRC) (61). Elevated levels of HSP90 and CALR on the surface of neoplastic cells have been associated with clinical responses amongst 18 patients with relapsed indolent non-Hodgkin’s lymphoma treated with an autologous cancer cell-based vaccine (62). Moreover, CALR exposure by malignant blasts has been linked to prolonged relapse-free (but not overall) survival in a cohort of 20 individuals with acute myeloid leukemia (AML) (63). Of note, the blasts of some of these patients exposed CALR spontaneously, and this correlated not only with the degree of EIF2A phosphorylation in malignant cells, but also with the ability of autologous T cells to secrete IFNγ on stimulation (63). Along similar lines, healthy individuals have been shown to differ from lung carcinoma patients with respect to the circulating levels of soluble CALR, as well as to the amount of CALR expressed on the surface of pulmonary (normal *versus* malignant) cells (64). Moreover, increased concentrations of soluble HSP90 have been detected in the serum of CRC patients (*n* = 172) as compared to healthy individuals (*n* = 10) (65). Interestingly, soluble HSP90 appears to activate cancer cell-intrinsic signaling pathways that promote disease progression (65, 66). These data indicate that cancer cells expose and/or shed CALR as well as HSPs even in the absence of chemotherapy (at least to some degree), possibly as a result of oncogenic stress and/or adverse microenvironmental conditions. Moreover, they suggest that membrane-bound CALR and HSPs have a different biological activity than their soluble counterparts.

**Table 1:** **Clinical studies assessing the prognostic and predictive value of ICD-associated CALR and HSP signaling in cancer patients**.

Parameter	Cancer	Treatment	No	Note(s)	Reference
CALR	AML	Anthracyclines-based chemotherapy	20	CALR exposure on blasts correlated with improved RFS	(63)
	Bladder carcinoma	Surgery	195	High CALR levels correlated with poor disease outcome	(67)
	Breast carcinoma	Surgery	23	High CALR levels correlated with poor MFS	(68)
	CRC	Surgical resection and chemotherapy	68	High CALR levels correlated with improved 5-y survival rate	(61)
	Gastric carcinoma	Gastrectomy and lymphadenectomy	79	High CALR levels correlated with poor disease outcome	(69)
	Lung carcinoma	n.a.	58	High CALR levels correlated with malignancy and tumor grade	(64)
		Radiotherapy	23	High CALR levels correlated with prolonged OS	(60)
	Mantle cell lymphoma	Surgery	163	High CALR levels correlated with poor disease outcome	(67)
	Neuroblastoma	Surgery alone or combined with chemotherapy	729 68	High CALR levels correlated with poor disease outcome High CALR levels correlated with favorable disease outcome	(67) (59)
	Non–Hodgkin’s lymphoma	Autologous cancer cell-based vaccine	18	CALR exposure was associated to clinical responses	(62)
	Ovarian carcinoma	Paclitaxel-based chemotherapy	220	High CALR levels correlated with prolonged DFS and OS	(60)

CD47	AML	n.a.	137	High CD47 levels correlated with shortened OS	(70)
	Esophageal carcinoma	Surgery	102	High CD47 levels correlated with shortened OS	(71)
	Ovarian carcinoma	Surgery	86	Low CD47 levels correlated with improved disease outcome	(72)

CD91	Melanoma	n.a.	16	High CD91 levels were associated with slow progression	(73)

ER stress	AML	Anthracycline-based chemotherapy	105	*XBP1* splicing correlated with prolonged DFS and OS	(74)
	Breast carcinoma	Anthracycline-based chemotherapy	60	Cancer cells from non-responders had high phosphorylation of EIF2A	(75)
		Surgical resection and/or hormonotherapy	100	*XBP1* splicing correlated with poor disease outcome	(76)
	DLBCL	Bortezomib	119	High HSPA5 levels correlated with worsened OS	(77)
	HNC	Surgery	79	High HSPA5 levels correlated with improved OS	(78)
	Lung cancer	Surgery	132	High HSPA5 levels correlated with improved disease outcome	(79)
	NSCLC	Surgery	193	PKR activation and EIF2A phosphorylation correlated with improved OS	(80)

HSP90	CRC	n.a.	182	Increased serum levels were associated with oncogenesis	(65)
	Non–Hodgkin’s lymphoma	Autologous cancer cell-based vaccine	18	CALR exposure was associated to clinical responses	(62)

HSPA1A	Gastric carcinoma	n.a.	39 patients	SNPs in *HSPA1A* affected disease incidence	(81)
			186 controls		

LMAN1	Ovarian carcinoma	n.a.	289 patients	SNPs in *LMAN1* affected disease incidence	(82)
			126 controls		

THBS1	Gastric carcinoma	n.a.	275 patients	SNPs in *THBS1* affected disease incidence	(83)
			275 controls		

*AML, acute myeloid leukemia; CRC, colorectal carcinoma; DFS, disease-free survival; DLBCL, diffuse large B-cell lymphoma; ER, endoplasmic reticulum; HNC, head and neck cancer; ICD, immunogenic cell death; MFS, metastasis-free survival; NSCLC, non-small cell lung carcinoma; n.a., not applicable or not available; OS; overall survival; RFS, relapse-free survival; SNP, single nucleotide polymorphism*.

Apparently at odds with the abovementioned clinical findings, total CALR levels have been positively associated with accelerated disease progression and poor outcome in a cohort of 79 gastric cancer patients (69), in 23 women with breast carcinoma upon surgery (68), as well in large cohorts of neuroblastoma (*n* = 729), bladder carcinoma (*n* = 195) and mantle cell lymphoma (*n* = 163) patients, irrespective of treatment type (67). Moreover, CALR expression by malignant cells failed to affect overall survival in 88 patients with esophageal squamous cell carcinoma treated with neo-adjuvant chemoradiotherapy and surgical resection (84). These results may reflect the intracellular functions of CALR in the preservation of reticular homeostasis, which is particularly important for malignant cells owing to their highly accelerated anabolic metabolism (85), or the fact that CALR exposure is generally associated with an increased expression of CD47, a very potent anti-phagocytic signal (67).

The phosphorylation of EIF2A as well as the activation of eukaryotic translation initiation factor 2-alpha kinase 2 (EIF2AK2, best known as PKR) have been associated with favorable disease outcome in a cohort of 193 non-small cell lung carcinoma (NSCLC) patients (80). On the contrary, elevated degrees of EIF2A phosphorylation in neoplastic cells have been correlated with nuclear size (a surrogate marker of DNA content), preferential tumor infiltration by T_REG_ cells, and poor disease outcome in a cohort of 60 breast carcinoma patients treated with anthracycline-based chemotherapy and tested longitudinally (75). Other manifestations on an ongoing UPR have been ascribed with prognostic or predictive value, including (but not limited to): (1) the expression levels of the ER chaperone heat shock 70 kDa protein 5 (HSPA5, best known as GRP78), as demonstrated in cohorts of 132 lung carcinoma patients (79), 79 individuals with head and neck cancer (78) and 119 patients with diffuse large B-cell lymphoma treated with the proteasome inhibitor bortezomib (which is a *bona fide* ICD inducer) (77); and (2) the splicing of X-box binding protein 1 (*XBP1*) (48), as demonstrated in a cohort of 105 AML patients tested at diagnosis (74). Of note, both CALR and GRP78 expression levels are also indirect manifestations of the activation of another branch of the ER stress response, *i.e*., the derepression of activating transcription factor 6 (ATF6) (74, 86). Finally, some studies have associated markers of an ongoing UPR with dismal disease outcome. For instance, Davies and colleagues have linked low levels of unspliced *XBP1* as well as a high spliced/unspliced *XBP1* ratio with poor disease outcome in 100 primary breast carcinoma patients treated with adjuvant hormonal therapy (76). The apparent discrepancy in these observations may reflect the differential reliance of distinct tumor types (or similar tumors at distinct stages of progression) on the ER stress response for survival in adverse microenvironment conditions (87).

Other processes and parameters linked to CALR and/or HSP exposure and their immunostimulatory effects have been shown to influence disease outcome in cancer patients. For instance, high CD47 levels have been reported to constitute an independent negative prognostic factor in cohorts of 86 patients with ovarian clear cell carcinoma (72), 102 individuals with esophageal squamous cell carcinoma (71), and 137 subjects with karyotypically normal AML (70). Along similar lines, the monocytes of 8 advanced melanoma patients progressing in an unusually slow fashion have been found to express increased amounts of CD91 as compared to those of 8 patients progressing normally (73). Moreover, single nucleotide polymorphisms (SNPs) affecting *HSPA1A* have been linked to an increased incidence of gastric carcinoma (as determined in a cohort of 39 patients and 186 controls) (81), a SNP affecting *THBS1* has been correlated with gastric cancer occurrence and progression in a cohort of 275 patients and 275 healthy individuals (83), while a SNP in *LMAN1* as well as the consequent decrease in LMAN1 levels appear to be associated with an increased risk for ovarian carcinoma (as determined in a cohort of 289 women seen in gynecologic oncology practice and 126 healthy volunteers) (82).

The robust immunostimulatory activity of HSPs has been harnessed to develop various anticancer vaccines that are nowadays in clinical development. These preparations generally consist in HSP-enriched (autologous or heterologous) cancer cell lysates that are administered directly to patients, in the presence of adequate immunological adjuvants (88, 89). The most common of these approaches relies on heat shock protein 90 kDa beta (Grp94), member 1 (HSP90B1, best known as GP96) and is often referred to as HSPPC-96 (Oncophage^®^ or Vitespen^®^) (90). So far, the safety and clinical profile of HSPPC-96 have been tested in cohorts of patients with metastatic melanoma (*n* = 36–322) (91–94), CRC (*n* = 29) (95), non-Hodgkin’s lymphoma (*n* = 20) (96); pancreatic adenocarcinoma (*n* = 10) (97), metastatic renal cell carcinoma (*n* = 84–409) (98, 99), glioma (*n* = 12) (100), recurrent glioblastoma (*n* = 41) (101), and assorted advanced malignancies (*n* = 16) (102). These studies demonstrate that the administration of HSPPC-96 to cancer patients is safe and is generally associated with markers of immunostimulation. However, most often such effects are weak and unable to mediate long-term therapeutic activity (99). Thus, further studies are required for translating the well-established ability of HSPs to stimulate the priming of TAA-specific immune responses into a therapeutic reality.

Taken together, these clinical observations suggest that CALR, HSPs and various processes associated with their exposure, secretion and signaling functions may have prognostic, predictive and therapeutic value.

## Type I IFN and TLR3 Signaling

Cancer cells responding to anthracyclines secrete type I IFNs as a consequence of TLR3 activation (39), and this is required for cell death to initiate adaptive immunity (39). By binding to homodimeric or heterodimeric receptors expressed on several immune effector cells, type I IFNs mediate multipronged immunostimulatory effects (40). In particular, type I IFNs promote cross-priming (103), boost the cytotoxic functions of CTLs and NK cells (104), and increase the survival of memory CTLs (105). Moreover, type I IFNs can protect antigen-activated CD8^+^ CTLs from elimination by NK cells (106, 107), trigger the secretion of pro-inflammatory mediators by macrophages (108), and counteract the immunosuppressive functions of T_REG_ cells (109). Besides such immunostimulatory effects, type I IFNs can ignite a cancer cell-intrinsic signal transduction pathway leading, amongst various effects, to the synthesis of the chemotactic factor chemokine (C–X–C motif) ligand 10 (CXCL10) (39). Indeed, at odds with their wild-type counterparts, *Ifnar1^−/−^* cancer cells succumbing to anthracyclines are unable to prime adaptive immune responses, even upon inoculation in wild-type hosts (39). Thus, type I IFN signaling in cancer cells appears to be critical for anthracycline-induced cell death to be perceived as immunogenic (39). Conversely, the efficacy of other immunotherapeutic agents such as the TLR7 agonist imiquimod requires type I IFN signaling in the host (110).

So far, only a few studies addressed the prognostic or predictive value of parameters reflecting the proficiency or activation status of TLR3 or type I IFN signaling (Table 2). High expression levels of TLR3 and/or toll-like receptor adaptor molecule 1 (TICAM1, a component of the TLR3 signaling apparatus best known as TRIF) have been associated with improved disease outcome in two cohorts of 85 and 172 subjects with hepatocellular carcinoma (HCC) (111, 112), as well as amongst 99 patients with neuroblastoma (113). Along similar lines, TLR3 expression levels have been shown to predict the response of 194 breast carcinoma patients treated with adjuvant radiotherapy plus a TLR3 agonist (114). SNPs affecting *TLR3* have been shown to influence prognosis in cohorts of 582 patients with CRC, especially among untreated individuals with Stage II disease (115) and 568 NSCLC patients (116). Along similar lines, *TLR3* SNPs have been associated with an altered risk for cervical cancer amongst 330 Tunisian women (117), breast carcinoma amongst 174 African-American women (118), oral squamous cell carcinoma amongst 197 individuals (119) HCC amongst 948 subjects (120), and CRC amongst more than 5,000 individuals (121). A type I IFN-related transcription signature centered around the expression of MX dynamin-like GTPase 1 (*MX1*) has been shown to predict the likelihood of 50 breast carcinoma patients to respond to neo-adjuvant anthracycline-based chemotherapy (39). Moreover, SNPs affecting interferon (alpha, beta and omega) receptor 1 (*IFNAR1*) have been associated with an increased risk for the development of CRC amongst 2085 individuals (122), as well as with significantly reduced overall survival in a cohort of 304 glioma patients (123). Similar results have been obtained for SNPs affecting the genes coding for two variants of IFNα (*i.e*., IFNA7 and IFNA8) (122, 123).

**Table 2:** **Clinical studies assessing the prognostic and predictive value of TLR3 status and type I IFN signaling in cancer patients**.

Parameter	Cancer	Treatment	No	Note(s)	Reference
IFNAR1	CRC	n.a.	1327 patients	A SNP in *IFNAR1* was linked to increased risk for oncogenesis	(122)
			758 controls		
	Glioma	n.a.	304	A SNP in *IFNAR1* was shown to affect patient OS	(123)

TLR3	Breast carcinoma	n.a.	102 patients	A SNP in *TLR3* was linked to increased risk for oncogenesis	(118)
			72 controls		
		polyA:U plus radiotherapy	194	High TLR3 levels predicted clinical responses to therapy	(114)
	Cervical carcinoma	n.a.	130 patients	A SNP in *TLR3* was linked to increased risk for oncogenesis	(117)
			200 controls		
	CRC	n.a.	582	SNPs in *TLR3* were shown to influence disease outcome	(115)
			2309 patients	SNPs in *TLR3* were linked to increased disease incidence	(121)
			2915 controls		
	HCC	n.a.	466 patients	A SNP in *TLR3* was linked to increased risk for oncogenesis	(120)
			482 controls		
			172	High TLR3 levels correlated with prolonged OS	(111)
		Surgery	85	High TLR3 levels correlated with prolonged OS	(112)
	Neuroblastoma	n.a.	99	High TLR3 levels correlated with favorable disease outcome	(113)
	NSCLC	Surgery	568	SNPs in *TLR3* were shown to influence disease outcome	(116)
	Oral squamous cell carcinoma	n.a.	93 patients	SNPs in *TLR3* were linked to increased risk for oncogenesis	(119)
			104 controls		
			240 patients	A SNP in *TLR4* was linked to increased risk for oncogenesis	(124)
			223 controls		

TRIF	HCC	Surgery	85	High TRIF levels correlated with prolonged OS	(112)

Type I IFN	Breast carcinoma	Anthracycline-based chemotherapy	50	A type I IFN-related signature predicted improved disease outcome	(39)
	CRC	n.a.	483	A SNP in *IFNA7* was shown to affect patient OS	(122)
	Glioma	n.a.	304	A SNP in *IFNA8* was shown to affect patient OS	(123)

*CRC, colorectal carcinoma; HCC, hepatocellular carcinoma; NSCLC, non-small cell lung carcinoma; n.a., not applicable or not available; OS; overall survival; SNP, single nucleotide polymorphism*.

The results of these studies suggest that monitoring biomarkers of TLR3 and type I IFN signaling may not only have prognostic/predictive relevance for cancer patients, but also inform on the risk for cancer development in healthy subjects. Of note, recombinant IFN-α2a (Roferon-A^®^) is approved by the US Food and Drug Administration and other regulatory agencies worldwide for use in subjects with hairy cell leukemia and Philadelphia chromosome-positive chronic myelogenous leukemia upon minimal pretreatment, while recombinant IFN-α2b (Intron A^®^) is currently employed for the treatment of hairy cell leukemia, AIDS-related Kaposi’s sarcoma, follicular lymphoma, multiple myeloma, melanoma, condyloma acuminata and cervical intraepithelial neoplasms.(125, 126) It remains to be determined to which extent, if any, the therapeutic efficacy of type I IFNs reflects their ability to promote the initiation of adaptive immune responses against dying cancer cells.

## Extracellular ATP and Autophagy

ATP is secreted during ICD through a mechanism that involves pannexin 1 (PANX1) channels and lysosomal exocytosis (127, 128). Importantly, autophagy is required for cancer cells succumbing to anthracyclines to release ATP in immunostimulatory amounts (42, 129, 130). Thus, the ability of anthracyclines to cause *bona fide* ICD is lost when cancer cells are rendered autophagy-deficient by genetic manipulations or engineered to overexpress ectonucleoside triphosphate diphosphohydrolase 1 (ENTPD1, best known as CD39), an enzyme that degrades extracellular ATP (42, 129). In line with this notion, the administration of CD39 inhibitors or CD39-neutralizing monoclonal antibodies reportedly relieves tumor-mediated immunosuppression (131), and (at least in some models) allows autophagy-deficient cells treated with anthracyclines to elicit normal immune responses upon inoculation in immunocompetent mice (42, 129). Extracellular ATP exerts immunostimulatory functions via at least three mechanistically distinct pathways: (1) by promoting the recruitment of APCs or APC precursors to sites of cell death, upon binding to purinergic receptor P2Y, G-protein coupled, 2 (P2RY2) (132–134); (2) by activating the so-called NLRP3 inflammasome and hence triggering the secretion of pro-inflammatory IL-1β (135, 136), an effect that relies on purinergic receptor P2X, ligand gated ion channel, 7 (41); and (3) by boosting the proliferation and cytotoxic activity of NK cells (26). Notably, extracellular ATP is sequentially metabolized by CD39 and 5′-nucleotidase, ecto (NT5E, best known as CD73) into ADP, AMP and adenosine, the latter of which has robust immunosuppressive effects (137).

Accumulating clinical evidence ascribes to parameters linked to the capacity of cancer cells to recruit and activate immune effectors (through extracellular ATP) a prognostic or predictive value for cancer patients (Table 3). A SNP compromising the function of P2RX7 has been associated with decreased time-to-metastasis in a cohort of 225 breast carcinoma patients treated with adjuvant anthracycline-based chemotherapy (41), with worsened clinicopathological parameters amongst 121 subjects with papillary thyroid cancer (138), and with an increased risk for the development of chronic lymphocytic leukemia (CLL), as determined in a cohort of 40 patients and 46 age-matched healthy individuals (139). Contrasting with these latter findings, however, the same SNP has been associated with increased overall survival in a cohort of 170 subjects with CLL (140), or found to have no correlation with disease incidence and/or outcome in independent cohorts of 144 CLL patients and 348 healthy controls (141), 121 individuals with CLL (142) 111 CLL patients and 97 controls (143), and 136 subjects with multiple myeloma (144). These apparently discrepant observations may reflect the cancer cell-intrinsic functions of P2RX7, which is known to control proliferation and regulated cell death (145). Of note, increased *P2RY2* mRNA levels have also been detected in gastric cancer biopsies from 14 patients (as compared to the adjacent healthy mucosa) (146), but these findings do not allow to determine whether gastric neoplasms were infiltrated by P2RY2^+^ immune cells or whether they overexpressed P2RY2.

**Table 3:** **Clinical studies assessing the prognostic and predictive value of ATP release and extracellular ATP signaling in cancer patients**.

Parameter	Cancer	Treatment	No	Note(s)	Reference
Autophagy	Breast carcinoma	n.a.	1067 patients	Low BECN1 levels correlated with worsened disease outcome	(147)
			1992 patients		
	HCC	Surgery	190	High LC3 levels correlated with prolonged OS	(148)
	HNC	Surgery	79	High LC3 levels correlated with node involvement and TNM score	(78)
	Pancreatic carcinoma	Surgery	73	High levels of BECN1 and other autophagy-related proteins correlated with poor outcome	(149)

CD39	CLL	n.a.	34 patients	High CD39 levels on T cells correlated with late disease	(150)
			31 controls		
			62	High CD39 levels on T cells correlated with late disease	(151)
	Endometrial cancer	Surgery	29	High CD39 levels correlated with tumor grade	(152)
	Pancreatic carcinoma	Surgery	28	High CD39 levels were linked to improved disease outcome	(153)

CD73	Endometrial cancer	Surgery	29	High CD73 levels correlated with tumor grade	(152)
	Glioblastoma	n.a.	500	CD73 downregulation was associated with improved DFS	(154)

P2RX7	Breast carcinoma	Anthracycline-based chemotherapy	225	A SNP in *P2RX7* was linked to shortened MFS	(41)
	CLL	n.a.	40 patients	A SNP in *P2RX7* was linked to increased risk for oncogenesis	(139)
			46 controls		
			144 patients	Lack of correlation between *P2RX7* status and disease incidence	(141)
			348 controls		
			111 patients	Lack of correlation between *P2RX7* status and disease incidence	(143)
			97 controls		
			170	A SNP in *P2RX7* was associated to increased OS	(140)
			121	Lack of correlation between *P2RX7* status and pathological features	(142)
	Multiple myeloma	n.a.	136 patients	Lack of correlation between *P2RX7* status and disease incidence	(144)
			95 controls		
	Papillary thyroid cancer	n.a.	121	A SNP in *P2RX7* was linked to poor clinicopathological features	(138)

P2RY2	Gastric cancer	n.a.	14 patients	Increased expression of P2RY2 in malignant cells	(146)

*CLL, chronic lymphocytic leukemia; DFS, disease-free survival; HCC, hepatocellular carcinoma; HNC, head and neck cancer; MFS, metastasis-free survival; n.a., not applicable or not available; OS; overall survival; SNP, single nucleotide polymorphism*.

Further corroborating the advantage conferred to malignant cells by an increased ability to convert immunostimulatory extracellular ATP into immunosuppressive AMP and adenosine, several studies ascribed a negative prognostic or predictive value to increased CD39 or CD73 levels. For instance, elevated amounts of CD39 and CD73 have been detected in 29 endometrial tumor samples as compared to the adjacent non-malignant tissues, and expression levels correlated with tumor grade (152). Along similar lines, CD39 (but not CD73) levels on the surface of CD4^+^ and CD8^+^ T cells have been shown to positively correlate with disease stage in two independent cohorts of 34 and 62 patients with CLL (150, 151), while CD73 downregulation has been associated with prolonged disease-free survival amongst 500 individuals with glioblastoma (154). At stark contrast with these findings, high levels of *CD39* mRNA have been linked to improved disease outcome in a cohort of 28 pancreatic cancer patients treated with surgery (153). The reasons underlying this discrepancy have not yet been clarified.

Of note, quantifying functional autophagy in tissue biopsies is rather complex, because most autophagic markers accumulate both when the autophagic flux is increased and when lysosomal degradation is blocked (155). Moreover, autophagy often serves a dual role in the course of tumor progression: (1) on the one hand it favors the survival of cancer cells exposed to adverse microenvironmental conditions (including nutritional, metabolic and therapeutic cues); (2) on the other hand, it is required for ICD-associated ATP secretion and for the elicitation of robust TAA-targeting immune responses (130, 156, 157). Notwithstanding these caveats, immunohistochemistry has been employed to study the prognostic or predictive value of autophagic markers such as the expression and lipidation of microtubule-associated protein 1 light chain 3 (MAP1LC3, best known as LC3) (158), with mixed results. For instance, LC3 expression has been associated with prolonged overall survival in a cohort of 190 HCC patients (148), but with lymph node involvement and high TNM score amongst 79 individuals with head and neck cancer (78). Along similar lines, reduced expression of beclin 1 (BECN1), a key component of the molecular machinery for autophagy, has been associated with poor prognosis in two independent cohorts of 1067 and 1992 breast carcinoma patients (147), but with improved disease outcome in a cohort of 73 patients with pancreatic cancer (149). These are only two examples of an abundant scientific literature correlating the expression of autophagy proteins in biopsies from patients affected with virtually all types of malignancies to clinicopathological features and/or markers of disease progression. The development of assays to monitor the functionality of the autophagic apparatus in clinical samples is urgently awaited to properly assess the prognostic and predictive value of autophagy for cancer patients.

## HMGB1 and Cell Death

According to current models, HMGB1 gets released in the course of cell death passively, upon the breakdown of the nuclear and plasma membrane (145, 159). Thus, besides differences in expression level, the extent of HMGB1 release generally correlates with the degree of cell death (160). However, changes in the oxidation status of extracellular HMGB1 have been suggested to dramatically alter its biological activity (161–163). Indeed, while reduced HMGB1 efficiently dimerizes with CXCL12 and mediate potent chemotactic functions upon binding to chemokine (C–X–C motif) receptor 4 (CXCR4) (164, 165), its oxidized counterpart fails to do so (162). Rather, oxidized HMGB1 signal via TLR2, TLR4 and advanced glycosylation end product-specific receptor (AGER, best known as RAGE) to stimulate the production of pro-inflammatory cytokines (162, 166–168). In addition, TLR4 signaling promotes cross-priming by inhibiting the fusion of antigen-containing endosomes with lysosomes (169). Interestingly, HMGB1 also binds to TLR9 (170) and hepatitis A virus cellular receptor 2 (HAVCR2, best known as TIM-3) (171), in particular when complexed with DNA. However, while TLR9 promotes cytokine secretion by plasmacytoid DCs and B cells (170), TIM-3 signaling blunts the ability of DCs to respond efficiently to inflammatory stimuli (171). Thus, extracellular HMGB1 mediates multipronged and context-dependent immunomodulatory functions.

Various clinical studies indicate that monitoring parameters linked to HMGB1 release and signaling may convey prognostic or predictive information for cancer patients (Table 4). High expression levels of HMGB1 in malignant cells have been shown to correlate with improved overall survival in 88 patients with esophageal squamous cell carcinoma subjected to neo-adjuvant chemoradiotherapy and surgical resection (84), as well as in 76 subjects with reseactable gastric adenocarcinoma (172). In a cohort of 232 breast carcinoma patients treated with anthracycline-based adjuvant chemotherapy, loss of nuclear HMGB1 has been positively associated with tumor size (173). Along similar lines, the co-expression of HMGB1 in the nucleus and in the cytoplasm of malignant cells has been shown to inversely correlate with tumor infiltration by CD45RO^+^ memory T cells and 5-year survival rate in 72 individuals with Stage IIIB CRC (174). Finally, HMGB1 overexpression has been shown to correlate with advanced clinical stage or decreased disease-free and/or overall survival amongst 164 patients with bladder carcinoma (175), 166 individuals with nasopharyngeal carcinoma (176), 192 CRC patients (177), 208 and 161 individuals with HCC (178, 179), 103 subjets with head and neck squamous cell carcinoma (180), as well as 85 patients with prostate cancer (181).

**Table 4:** **Clinical studies assessing the prognostic and predictive value of HMGB1 release and extracellular HMGB1 signaling in cancer patients**.

Parameter	Cancer	Treatment	No	Note(s)	Reference
CASP3	Endometrial carcinoma	n.a.	1028 patients	A SNP in *CASP3* was linked to increased risk for oncogenesis	(182)
			1003 controls		

CASP7	Endometrial carcinoma	n.a.	1028 patients	SNPs in *CASP7* were linked to increased risk for oncogenesis	(182)
			1003 controls		

CASP9	CRC	n.a.	402 patients	SNPs in *CASP9* were linked to decreased risk for oncogenesis and improved disease outcome	(183)
			480 controls		

HMGB1	Bladder carcinoma	n.a.	164	High HMGB1 levels correlated to worsened disease outcome	(175)
	Breast carcinoma	Anthracycline-based chemotherapy	232	Loss of nuclear HMGB1 positively correlated with tumor size	(173)
			41	Increases in circulating HMGB1 were linked to clinical response	(184)
	CRC	n.a.	219 patients	High levels of serum HMGB1 correlated with disease incidence	(185)
			75 controls		
		n.a.	192	High HMGB1 levels correlated with worsened disease outcome	(177)
		Radioembolization therapy	49	High levels of serum HMGB1 correlated with decreased OS	(186)
		Surgery	72	Co-expression of HMGB1 in the nucleus and in the cytoplasm of malignant cells was linked to worsened 5-year survival rate	(174)
	Esophageal carcinoma	Chemoradiotherapy and surgery	88	High HMGB1 levels correlated with improved OS	(84)
	Gastric adenocarcinoma	Surgery	76	High HMGB1 levels in malignant cells correlated with improved OS	(172)
	HCC	n.a.	208	High HMGB1 levels correlated with worsened disease outcome	(179)
			161	High HMGB1 levels correlated with worsened disease outcome	(178)
	HNC	n.a.	71 patients	High levels of serum HMGB1 correlated with disease progression	(187)
			50 controls		
			103	High HMGB1 levels correlated with worsened disease outcome	(180)
	Malignant mesothelioma	n.a.	61 patients	High levels of serum HMGB1 correlated with disease incidence	(188)
			45 controls		
	Nasopharyngeal carcinoma	n.a.	166	High HMGB1 levels correlated with worsened disease outcome	(176)
	Pancreatic carcinoma	Multicomponent chemotherapy	78	High circulating HMGB1 correlated with poor therapy response	(189)
		n.a.	70	High levels of serum HMGB1 correlated with decreased OS	(190)
	Prostate carcinoma	n.a.	85	High HMGB1 levels correlated with worsened disease outcome	(181)
	Solid tumors	Virotherapy	17	Increases in circulating HMGB1 levels were linked to clinical response	(191)
			202	Increases in circulating HMGB1 levels were linked to clinical response	(192)

MYD88	CRC	Surgery	108	High MYD88 levels correlated with shortened DFS and OS	(193)
	Lymphoma	Conventional chemotherapy	29	MYD88 mutations were involved in the pathogenesis of the disease	(194)
	Ovarian carcinoma	Surgery	123	High MYD88 levels correlated with worsened disease outcome	(195)
			109	High MYD88 levels correlated with shortened DFS and OS	(196)

RAGE	Breast carcinoma	n.a.	509 patients	A SNP in *AGER* was linked to increased risk for oncogenesis	(197)
			504 controls		
			120 patients	High levels of circulating RAGE correlated with advanced disease stage but improved outcome	(198)
			92 controls		
	Gastric carcinoma	Surgery	180	High RAGE levels were associated with worsened disease outcome	(199)
	HCC	Transarterial chemoembolization	71	High levels of circulating RAGE correlated with clinical response	(200)
	NSCLC	Platinum-based chemotherapy	562 patients	SNPs in *AGER* were linked to increased risk for oncogenesis and differential clinical response	(201)
			764 controls		
	Ovarian carcinoma	n.a.	190 patients	A SNP in *AGER* was linked to increased risk for oncogenesis	(202)
			210 controls		

TLR2	CRC	n.a.	2309 patients	SNPs in *TLR2* were associated with decreased 5-year survival rate	(121)
			2915 controls		
	Gastric carcinoma	n.a.	289 patients	A SNP in *TLR2* was linked to increased risk for oncogenesis	(203)
			400 controls		
	HCC	n.a.	211 patients	SNPs in *TLR2* were linked to increased risk for oncogenesis	(204)
			232 controls		
	Lymphoma	n.a.	710 patients	A SNP in *TLR2* was linked to increased risk for oncogenesis	(205)
			710 controls		
	Prostate carcinoma	n.a.	195 patients	A SNP in *TLR2* was linked to increased risk for oncogenesis	(206)
			250 controls		

TLR4	Breast carcinoma	Anthracycline-based chemotherapy	280	A SNP in *TLR4* was linked to shortened MFS	(43)
	CRC	n.a.	2309 patients	SNPs in *TLR4* were associated with risk variations and increased OS	(121)
			2915 controls		
		Surgery	108	High TLR4 levels were associated with shortened DFS and OS	(193)
	HNC	Adjuvant systemic chemotherapy	188	A SNP in *TLR4* was linked to shortened DFS and OS	(207)
	Melanoma	Allogenic cancer cell-based vaccine	72	A SNP in *TLR4* was linked to shortened DFS and OS	(208)
		Various	622	A SNP in *TLR4* was linked to shortened DFS and OS	(209)
	Ovarian carcinoma	Surgery	123	High TLR4 levels were associated with worsened disease outcome	(195)
	Prostate carcinoma	n.a.	700 patients	A SNP in *TLR4* was linked to increased risk for oncogenesis	(210)
			700 controls		
			258 patients	A SNP in *TLR4* was linked to increased risk for oncogenesis	(211)
			258 controls		
			157 patients	A SNP in *TLR4* was linked to increased risk for oncogenesis	(212)
			143 controls		
			240 patients	A SNP in *TLR4* was linked to increased risk for oncogenesis	(124)
			223 controls		

*CRC, colorectal carcinoma; DFS, disease-free survival; HCC, hepatocellular carcinoma; HNC, head and neck cancer; MFS, metastasis-free survival; NSCLC, non-small cell lung carcinoma; n.a., not applicable or not available; OS; overall survival; RFS, relapse-free survival; SNP, single nucleotide polymorphism*.

Notably, circulating HMGB1 and RAGE levels have been intensively investigated for their predictive or prognostic value. Elevations of HMGB1 in the serum have been correlated with incidence, progression or unfavorable disease outcome in cohorts of 49 individuals with CRC, or 219 CRC patients and 75 healthy controls (185, 186), 70 individuals with pancreatic adenocarcinoma (190), 71 laryngeal squamous cell carcinoma patients and 50 healthy controls (187), 61 subjects with malignant pleural mesothelioma (188), and 78 pancreatic carcinoma patients (189). Conversely, a treatment-related increase in the circulating levels of HGMB1 has been associated with pathological complete response or partial remission amongst 41 breast carcinoma patients treated with neo-adjuvant chemotherapy based on epirubicin (an ICD inducer) (184), as well as amongst 17 and 202 subjects with chemotherapy-refractory tumors treated with oncolytic virotherapy (191, 192). High levels of RAGE in the serum have been linked to advanced tumor stage but improved clinical outcome amongst 120 patients with breast carcinoma (198). Along similar lines, serum RAGE concentrations were significantly higher in 32 individuals with HCC who favorably responded to transarterial chemoembolization therapy than in 39 patients who progressed upon treatment (200).

Thus, in many (but not all) clinical settings high intratumoral and circulating levels of HMGB1 have a negative prognostic or predictive value. These findings may reflect the ability of some tumors to retain HMGB1 in the course of stress response, the intrinsic resistance of such tumors to the induction of cell death, or the cancer cell-intrinsic functions of HMGB1 (213). In other settings, however, circulating HMGB1 and RAGE levels appear to reflect well the death of cancer cells exposed to immunogenic treatment modalities (184, 191, 192). Possibly, the timing of detection plays a critical role in this setting, calling for the development of optimized monitoring procedures.

SNPs in *TLR2*, *TLR4* and *AGER*, as well as the circulating levels of a soluble RAGE variant have been shown to affect cancer susceptibility as well as disease outcome in several studies. In particular, *TLR2* polymorphisms have been linked to an increased risk for lymphoma (as determined in 710 patients and as many healthy subjects) (205), gastric carcinoma (as assessed in 289 patients and more than 400 controls) (203), prostate carcinoma (as investigated in 195 patients and 250 healthy individuals) (206), HCC (as tested in 211 patients and 232 controls) (204), and CRC (as assessed in 2,309 patients and 2,915 healthy individuals) (121). Loss-of-function variants of TLR4 have been associated with decreased time-to-metastasis amongst 280 women with non-metastatic breast carcinoma treated with surgery followed by anthracycline-based chemotherapy and local irradiation (43), with reduced disease-free and overall survival amongst 188 head and neck cancer patients receiving adjuvant systemic therapy (207), amongst 72 melanoma patients vaccinated with a heat-shocked allogeneic melanoma cell line (208), and amongst 622 melanoma patients subjected to various treatment modalities (209). Along similar lines, SNPs affecting *TLR4* or *AGER* have been linked to an increased risk for prostate cancer (as determined in multiple studies collectively testing more than 1,000 patients and as many age-matched controls) (124, 210–212), ovarian cancer (as assessed in a study testing 190 patients and 210 controls) (202), breast carcinoma (as investigated in 509 patients and 504 healthy women) (197), CRC (as determined in a large cohort encompassing 2,309 patients and 2,915 healthy individuals) (121), and NSCLC (as tested in 562 patients and 764 controls) (201). Notably, this latter study also identified a specific *AGER* SNP associated with a differential response of NSCLC patients to chemotherapy (201).

Conversely, elevated expression levels of RAGE, TLR4 and/or components of the TLR signaling machinery like myeloid differentiation primary response gene 88 (MYD88) by malignant tissues have been correlated with shortened disease-free and overall survival in 2 cohorts of 109 and 123 ovarian carcinoma patients subjected to surgery (195, 196), in a cohort 108 subjects with CRC (193), and amongst 180 individuals with gastric carcinoma (199). Along similar lines, activating mutations in *MYD88* have been linked to the pathogenesis of primary central nervous system lymphomas (194). Most likely, these findings reflect the advantage conferred to malignant cells by the expression of RAGE and TLR4, which can activate robust pro-survival pathways via NF-κB (214).

Finally, distinct SNPs affecting caspase-7 (*CASP7*) and one affecting caspase-3 (*CASP3*) have been associated with an altered risk for endometrial carcinoma (as investigated in a cohort of 1,028 patients and 1,003 healthy women) (182), whereas SNPs affecting caspase-9 (*CASP9*) have been linked to reduced CRC incidence or improved disease outcome (as determined in a cohort of 402 patients and 480 healthy controls) (183). It remains to be determined whether these SNPs truly compromise the ability of cancer cells to emit DAMPs (and hence trigger immunosurveillance mechanisms).

## Other DAMPs

The abovementioned molecules and processes may constitute only the tip of an iceberg, meaning that several other DAMPs may contribute to the immunogenicity of cell death, at least in some circumstances. These DAMPs include (but are not limited to) various mitochondrial products like mtDNA, cardiolipin and *N*-formylated peptides (30) as well as cytosolic proteins like filamentous F-actin (45). Robust preclinical evidence implicates mtDNA in the etiology of septic and non-septic shock as well as in heart failure (29, 215). Cytosolic, extra-cytosolic and extracellular mtDNA molecules have indeed robust pro-inflammatory effects as they trigger type I IFN synthesis via transmembrane protein 173 (TM173, best known as STING) (216) or TLR9 activation (215). In line with this notion, circulating mtDNA levels have been shown to reflect the degree of inflammation and the extent of tissue damage in patients under maintenance hemodialysis (217). Moreover, mtDNA concentrations in the plasma of severe sepsis patients admitted to the emergency room have been ascribed robust predictive value on disease outcome (218). Upon binding to formyl peptide receptor 1 (FPR1), *N*-formylated peptides reportedly attract neutrophils, stimulate their degranulation, activate monocytes and favor the production of pro-inflammatory cytokines (219–223). Cardiolipin, a lipid that is specifically contained in the inner mitochondrial membrane, binds CD1D on the surface of APC, thus endowing them with the ability of priming CD1D-restricted γδ T cells (224). Finally, F-actin becomes accessible upon disruption of the plasma membrane and promotes the elicitation of adaptive immune responses against dead cell-associated antigens by binding to C-type lectin domain family 9, member A (CLEC9A, best known as DNGR1) on the surface of DCs (45). Studies elucidating the actual contribution of these DAMPs to ICD are urgently awaited.

## Concluding Remarks

**Figure 1:**
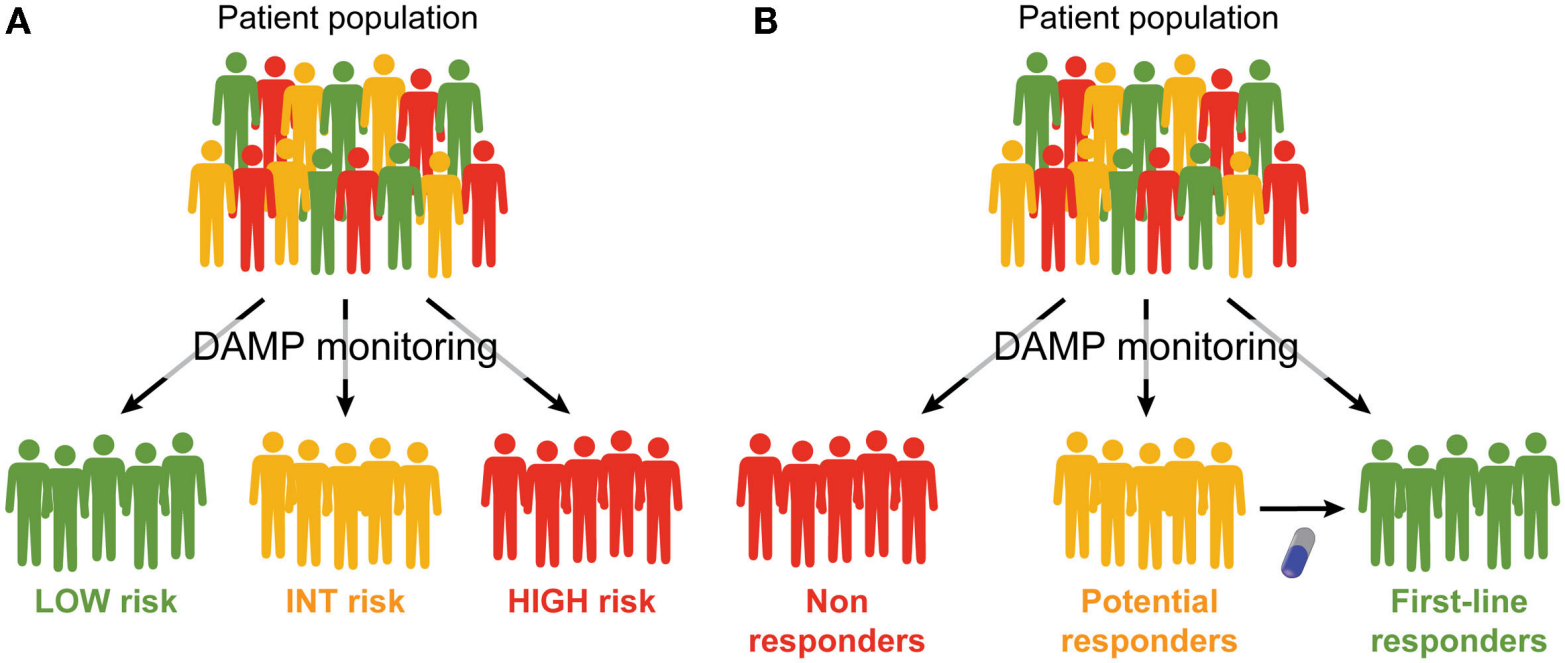
**Prognostic and predictive value of DAMPs and DAMP-associated processes**. **(A,B)**. Monitoring the emission of damage-associated molecular patterns (DAMPs) or DAMP-associated processes may have a multifaceted impact on the clinical management of cancer patients. First, it may allow for a prognostic assessment and permit the stratification of patients in different risk groups **(A)**. Second, it may allow for the identification of patients who are intrinsically capable or uncapable to respond to a specific treatment, and amongst the latter, those who may benefit from combinatorial therapeutic approaches aimed at restoring normal DAMP signaling **(B)**.

It is now clear that the emission of DAMPs according to a specific spatiotemporal pattern is an absolute requirement for the elicitation of immune responses against malignant cells succumbing to treatment, and that such responses are necessary for the full-blown efficacy of most (if not all) anticancer therapeutic regimens. In many settings, however, neoplastic cells exposed to conventional chemotherapeutics, radiotherapy or targeted anticancer agents fail to emit DAMPs in a manner compatible with the activation of the immune system, calling for the development of complementation strategies (16). Several approaches are being conceived to address this issue, including the implementation of combinatorial therapeutic regimens including (1) ER stressors, recombinant CALR or recombinant HSPs, to complement for defects in the CALR or HSP exposure pathway; (2) TLR3 agonists or recombinant type I IFNs, to correct problems in the secretion of type I IFN; (3) autophagy inducers or inhibitors of extracellular ATP-degrading enzymes, to maximize the amount of ATP secreted in the course of cell death; and (4) recombinant HMGB1, TLR4 agonists or cytotoxic agents, to restore HMGB1-dependent immunostimulation (225). Besides, consistent efforts are being devoted to the identification of additional strategies that *per se* induce ICD, *in vivo* (with direct therapeutic purposes), and *in vitro* (for instance, for the development of anticancer vaccines) (20). Monitoring DAMPs and DAMP-associated processes may therefore have a dual clinical relevance (Figure 1). First, it may improve patient stratification by allowing for the identification of individuals with different prognosis and/or subjects who are likely to respond (or are responding) to a particular therapeutic regimen. Second, it may instruct therapeutic choices by spotting specific molecular or cellular defects that may be corrected pharmacologically. We surmise that the prognostic and/or predictive value of DAMPs and DAMP-associated processes will have a significant impact on the clinical management of cancer patients.

## Conflict of Interest Statement

Jitka Fucikova, Irena Moserova, Linda Urbanova, and Radek Spisek are employees of Sotio (Prague, Czech Republic). The other co-authors declare that the research was conducted in the absence of any commercial or financial relationships that could be construed as a potential conflict of interest. The handling editor, Fabrizio Mattei declares that, despite having co-authored a paper with the manuscript’s authors within the past two years, the review process was handled objectively.

## Abbreviations

AGER, advanced glycosylation end product-specific receptor; AML, acute myeloid leukemia; APC, antigen-presenting cell; ATF6, activating transcription factor 6; BECN1, beclin 1; C1q, complement component 1, q subcomponent; CALR, calreticulin; CLEC9A, C-type lectin domain family 9, member A; CLL, chronic lymphocytic leukemia; CRC, colorectal carcinoma; CXCL10, chemokine (C-X-C motif) ligand 10; CXCR4, chemokine (C-X-C motif) receptor 4; DAMP, damage-associated molecular pattern; EIF2A, eukaryotic translation initiation factor 2A; EIF2AK2, eukaryotic translation initiation factor 2-alpha kinase 2; ENTPD1, ectonucleoside triphosphate diphosphohydrolase 1; FPR1, formyl peptide receptor 1; HCC, hepatocellular carcinoma; HMGB1, high mobility group box 1; HSP, heat-shock protein; HSP90AA1, heat shock protein 90 kDa alpha (cytosolic), class A member 1; HSPA1A, heat shock 70 kDa protein 1A; HSPA5, heat shock 70 kDa protein 5; HSP90B1, heat shock protein 90 kDa beta (Grp94), member 1; ICD, immunogenic cell death; IFN, interferon; IFNA8, interferon, alpha 8; IFNAR1, interferon (alpha, beta and omega) receptor 1; IL-6, interleukin-6; KLRD1, killer cell lectin-like receptor subfamily D; LMAN1, lectin, mannose-binding, 1; MAP1LC3, microtubule-associated protein 1 light chain 3; MX1, MX dynamin-like GTPase 1; MYD88, myeloid differentiation primary response gene 88; NSCLC, non-small cell lung carcinoma; NK, natural killer; NT5E, ecto 5′-nucleotidase; P2RY2, purinergic receptor P2Y, G-protein coupled, 2; PLSCR1, phospholipid scramblase 1; PS, phosphatidylserine; TAA, tumor-associated antigen; THBS1, thrombospondin 1; TICAM1, Toll-like receptor adaptor molecule 1; TLR, Toll-like receptor; TM173, transmembrane protein 173; TNFα, tumor necrosis factor α; UPR, unfolded protein response; XBP1, X-box binding protein 1.
